# Phrase Frequency Effects in Language Production

**DOI:** 10.1371/journal.pone.0033202

**Published:** 2012-03-27

**Authors:** Niels Janssen, Horacio A. Barber

**Affiliations:** Departamento de Psicología Cognitiva, Social y Organizacional, Universidad de La Laguna, La Laguna, Santa Cruz de Tenerife, Spain; University of Barcelona, Spain

## Abstract

A classic debate in the psychology of language concerns the question of the grain-size of the linguistic information that is stored in memory. One view is that only morphologically simple forms are stored (e.g., ‘car’, ‘red’), and that more complex forms of language such as multi-word phrases (e.g., ‘red car’) are generated on-line from the simple forms. In two experiments we tested this view. In Experiment 1, participants produced noun+adjective and noun+noun phrases that were elicited by experimental displays consisting of colored line drawings and two superimposed line drawings. In Experiment 2, participants produced noun+adjective and determiner+noun+adjective utterances elicited by colored line drawings. In both experiments, naming latencies decreased with increasing frequency of the multi-word phrase, and were unaffected by the frequency of the object name in the utterance. These results suggest that the language system is sensitive to the distribution of linguistic information at grain-sizes beyond individual words.

## Introduction

One of the more remarkable discoveries in the psychology of language is that word comprehension and production are affected by the frequency with which words appear in the language [1], [2]. This observation suggests that the language system is sensitive to the distribution of linguistic information in the language environment. A classic debate concerns the grain-size of the linguistic information to which the system is sensitive. The traditional view is that the grain size is rather restricted, and that the system is only sensitive to the distribution of morphologically simple forms [3], [4]. A contrasting proposal is that the grain-size extends beyond morphologically simple forms, and that the system is sensitive to the distribution of morphologically complex words, and multi-word phrases [5]–[15]. Much of the data that has been used to distinguish between these two views comes from the comprehension and production of morphologically complex words (see [16] for a review). Here we focused on the production of multi-word phrases. In the experiments reported below, participants produced two- and three-word phrases whose frequency of occurrence in the language varied.

These different predictions about the grain-size are derived from two fundamentally different views of the language system. The traditional view is that the language system consists of two separate parts: A mental lexicon in which linguistic elements are stored (i.e., *words*), and a formal grammar that interacts with the stored elements in the lexicon (i.e., *rules*; [3], [4]). The grammar is assumed to represent an innately specified set of formal rules that describes how to combine morphologically simple forms (e.g., ‘car’, ‘red’ ‘plural-s’) into more complex words (e.g., ‘cars’) and multi-word phrases (e.g., ‘red car’). The words and rules view strongly adheres to the principle of economy, meaning that only those linguistic forms are stored that cannot be computed by means of rules. Given the assumption that only morphologically simple forms are stored, it follows that the system can only keep track of the distributional information for such stored forms. Thus, according to the words and rules view, the language system should be sensitive to the distributional information of morphologically simple forms, but not of morphologically complex words and multi-word phrases.

Alternatively, one might consider a radically different view of the language system. Most critically, this view rejects a core aspect of the words and rules view – that the formal grammar is based on innately specified linguistic information. Instead, this new view emphasizes the role of experience in language learning, and assumes that a grammar emerges from a user's direct experience with the language. In other words, this view assumes that lexicon and grammar cannot be separated, and that a grammar will gradually emerge from the repeated exposure and analysis of complex linguistic forms. This view of the language system is central in recent connectionist [5]–[7], usage-based [8]–[11], and exemplar-based [12]–[15] approaches to language processing. Following others [17], we will refer to these models as *emergentist models* of the language system. In order to derive the grammar, emergentist models assume that the language system is sensitive to the distribution of linguistic information at grain-sizes larger than morphologically simple forms.

One way to distinguish between the words and rules and emergentist models is to consider how language comprehension and production latencies are affected by the frequency with which complex words and multi-word phrases appear in the language. Frequency of occurrence is an index of the experience that a language user has with a given linguistic token. As we mentioned above, there are frequency effects in the comprehension and production of morphologically simple words [1], [2]. The two models discussed above make different predictions about whether the comprehension and production of complex words and multi-word phrases should be sensitive to the frequency of their component parts (i.e., part-frequency), or whether they should be sensitive to the frequency of the complex word and multi-word phrases themselves (i.e., surface-frequency). According to the words and rules model, the system is only sensitive to the distribution of morphologically simple forms, and therefore predicts a part-frequency effect in the comprehension and production of morphologically complex and multi-word phrases. By contrast, according to the emergentist models, the system is sensitive to the distribution of complex words and multi-word phrases, and hence, predicts a surface frequency effect in the comprehension and production of morphologically complex words and multi-word phrases. Consider, for example, a multi-word phrase like ‘red car’. The two models disagree about whether comprehension and production latencies are sensitive to the frequency with which the component parts (i.e., ‘red’ and ‘car’) appear in the language (i.e., a part-frequency effect), or whether they are sensitive to the frequency with which the whole phrase (i.e., ‘red car’) appears in the language (i.e., a surface frequency effect).

Much of the research on this topic has focused on the comprehension and production of morphologically complex words. Specifically, these studies have addressed the question of whether comprehension and production latencies of morphologically complex words reveal a part- or a surface-frequency effect. For example, in Sereno and Jongman [18], participants were asked to decide whether a presented string was a word or not. Words could be presented in singular (e.g., ‘car’) or in plural (e.g, ‘cars’). If the language system were sensitive only to the distribution of morphologically simple forms, one would expect a part-frequency effect: Response latencies should be sensitive to the frequency of the singular form in both singular and plural presentation of the words. By contrast, if the language system were sensitive to the distribution of morphologically complex forms, one would expect a surface-frequency effect: Latencies should be sensitive to the frequency of the surface form of the word. In line with this latter prediction, the results revealed that decision latencies were sensitive to the frequency of the singular form when the word was presented in singular, and to the frequency of the plural form when the word was presented in plural. Comparable results have been found in the field of language production. Specifically, Janssen et al. [19] have shown that naming latencies to pictures with morphologically complex compound names (e.g., ‘doghouse’) were sensitive to the compounds' surface-word frequency, and not its part-frequency. These data suggest that the language processing system is sensitive to the distribution of linguistic information at grain-sizes beyond the individual word. Consequently, these data have formed the basis for an argument against the words and rules view of language processing [16].

A much smaller body of evidence has examined multi-word phrases, and the majority of studies have been conducted in a language comprehension context. In probably the most comprehensive study to date, Arnon and Snider [17] asked participants to decide whether a visually presented four-word phrase was a grammatically correct English phrase. Phrases were either low frequency (e.g., ‘I want to say’), or high frequency (e.g., ‘I want to go’). Within each phrase, individual word, bigram, and trigram frequencies were controlled. Importantly, sets of low and high frequency phrases were selected at three points along the frequency continuum (i.e., low, middle, and high). This manipulation was included to examine whether phrase frequency effects would be restricted to phrases with high average phrase frequencies (thereby suggesting a certain threshold for storage), or whether a phrase frequency effect would be found across the entire frequency continuum. The results from two experiments and a meta-analysis revealed that response latencies were sensitive to a factorial and continuous manipulation of the phrase frequency variable. These phrase frequency effects were found across the entire frequency continuum (i.e., at low, middle and high points) and were interpreted as challenging the words and rules model.

Likewise, Siyanova-Chanturia, Conklin, and van Heuven [20], asked speakers to read three-word binomial phrases (e.g., ‘bride and groom’) while registering their eye movements. The results revealed that various eye-movement measures (first pass reading time, total reading time, and fixation count) were sensitive to the frequency with which a token phrase appeared in the language. In addition, a study by Sosa and MacFarlane [21] revealed that the speed with which listeners were able to detect the word ‘of’ was determined by the frequency of the phrase in which the word appeared. Finally, Bannard and Matthews [22] have shown that two and three-year old infants are better at repeating and producing high (e.g., ‘sit in your chair’) versus low (e.g., ‘sit in your truck’) frequency phrases (see [23]–[25] for additional results). Thus, there now exists a substantial body of evidence that suggest that phrase frequency affects the processing of multi-word phrases in a language comprehension context. As with the evidence from the processing of complex words, these data suggest that the language system is sensitive to the distribution of linguistic information at grain-sizes beyond morphologically simple forms, and are therefore inconsistent with the assumptions of the words and rules model.

In the current study we attempted to show that phrase frequency affects the processing of multi-word phrases in a language production context. There are three reasons for investigating whether phrase frequency affects the production of multi-word phrases. First, there has been, to our knowledge, only one study that has examined the impact of phrase frequency on multi-word production, and it is therefore important to establish the reliability of this effect. Bybee and Scheibman [26] analyzed digitized productions of the word “don't” spoken in a variety of contexts in American English. Their study revealed that the degree of phonological reduction of this word was affected by the frequency of the phrase in which the word appeared. Specifically, the reduction was most pronounced in utterances that were the most frequent (e.g., “I don't know”), suggesting that phrase frequency affected the production of multi-word phrases. However, these results are inconclusive. The study focused only on the production of a single word (“don't”), and it is therefore not clear whether there is something special about phrases that contain this word, or whether these results generalize to other multi-word phrases. In addition, this study examined the impact of phrase frequency on phonetic duration measures, and it is not clear whether these results generalize to other dependent measures such as naming latencies.

A second reason for investigating whether phrase frequency impacts the production of multi-word phrases is that phrase frequency effects have been predominantly found in studies of visual comprehension, and it is therefore possible that the phrase frequency effect arises as a consequence of specific properties of the visual language comprehension system. For example, during reading, multiple words might be present in the input representation. Consequently, the phrase frequency effects found in comprehension studies might reflect a system for which it is beneficial to process linguistic information at grain-sizes beyond the individual word. By contrast, language production proceeds mostly on a word-by-word basis. Under such circumstances, the system would not need to process linguistic information at a grain-size beyond the individual word. Consequently, one might expect that the phrase frequency effects observed in comprehension studies would not be found in a language production context. Thus, establishing the phrase frequency effect in language production would rule out the possibility that the effect arises due to specific aspects of the language comprehension system.

Finally, there are clear theoretical motivations for investigating phrase frequency effects in language production. This is because traditional models of language production adhere to the words and rules view, and therefore do not predict phrase frequency effects in the production of multi-word phrases [27], [28]. Specifically, current production models assume that the lexicon contains only morphologically simple forms, and that complex words and multi-word phrases are generated by the application of rules. To illustrate, consider for example, the model of Dell [27]. In this model, word retrieval is driven by both semantic and syntactic information (and also to some extent by phonological information), where semantic information drives the initial activation of words in the lexicon, and syntactic information is used to constrain the order in which the words are selected and ordered for linear output. Crucially, this model assumes that only morphologically simple forms are stored in the lexicon, and that morphologically complex words and multi-word phrases are generated on-line from the combination of the morphologically simple forms. Thus, traditional models of language production adhere to the words and rules view, and assume that only morphologically simple forms are stored. Consequently, they predict no phrase frequency effects for multi-word phrases.

## Experiment 1

In the Experiment reported below we attempted to generalize the phrase frequency effects previously found in language comprehension studies, to a language production context. Specifically, native Spanish speakers produced noun+adjective and noun+noun phrases elicited by stimulus displays containing a colored object or two objects, respectively. The frequency of usage of the first noun response in the two phrase types, as well as the frequency of usage of the token phrase were manipulated. Based on previous research we expected to find an object name frequency effect in the production of these phrases [29]. In addition, if previous results obtained in language comprehension generalize to language production, we expected to find a phrase frequency effect. In line with other studies that have examined whether phrase frequency effects are limited to phrases selected from the high end of the frequency continuum, or whether phrase frequency effects are found across the entire range of phrase frequencies [17], we included two different phrase types (noun+adjective and noun+noun). The average phrase frequency of noun+adjective utterances is substantially higher than that of noun+noun utterances, given that in Spanish, nouns often are followed by an adjective, whereas a noun is not often followed by another noun. Noun+noun combinations generally appear in languages (including Spanish and English) as lists separated by punctuation marks (e.g., “… by car_N_, bus_N_, or …”), and have been used in multi-word production studies to test claims about the production system (e.g., [30]). Thus, comparing phrase frequency effects in these two phrase types will allow us to identify whether phrase frequency effects are found in limited frequency ranges or across the entire frequency range.

To anticipate the results, we found a phrase frequency effect in the production of both noun+noun and noun+adjective phrases. Experiment 2 served to replicate, and rule out two alternative interpretations of this effect.

### Materials and Methods

**Ethics Statement**: The Comité de Ética de la Investigación y de Bienestar Animal (CEIBA) of the University of La Laguna (the University's ethics committee), waived the need for the approval of this study, given that no confidential information was collected, the experiment did not induce a stressful situation, and did not involve negative, or emotionally adverse stimuli. All data were analyzed anonymously, and therefore it was not necessary for participants to provide informed consent. Participants were told they could terminate the experiment at any moment and at their own volition. All data were obtained according to the principles expressed in the Declaration of Helsinki.

**Participants**: The participants were twenty-six native speakers of Spanish, all students at the University of La Laguna. They received course credit for participation.

**Materials**: Fifty objects were selected from the International Picture Naming Project [31]. All objects had high name agreement (H<0.75; lower values of H indicate higher name agreement). These objects elicited the first response in the noun+adjective and noun+noun phrases. To elicit the second response in the noun+adjective condition, ten colors (rojo [red], verde [green], azul [blue], amarillo [yellow], naranja [orange], marron [brown], morado [purple], rosa [pink], gris [grey], negro [black]) were chosen. To elicit the second response in the noun+noun condition, ten additional objects (rodillo [rolling pin], volcán [volcano], avión [airplane], anillo [ring], niña [girl], mujer [woman], martillo [hammer], rana [frog], güante [glove], niño [boy]) were selected from the same database. These two sets of 10 color and object names were matched on phonological onset, and a given color-object pair was assigned to the same object corresponding to the first response. For example, the color-object pair ‘rojo’ and ‘rodillo’ were assigned to the same first response object ‘zapato’ to form the ‘zapato rojo’ and ‘zapato rodillo’ items. There was no phonological relationship between the onset of the first and second responses in the two phrase types. See Table S1 for an overview of the items.

We ensured that the color prototypicality of the objects in the noun+adjective phrases was unrelated to the manipulation of phrase frequency. Given that picture naming latencies depend on whether a given object is presented in its prototypical color [32], it was important to ensure that potential effects of phrase frequency were unrelated to effects of color prototypicality. Two specific measures were taken. First, all objects in the experiment were systematically assigned a non-prototypical color. This ensured that no object appeared in its prototypical color, thereby avoiding a correlation between an object's color prototypicality and phrase frequency. Second, although we avoided the selection of objects with a strong prototypical color, there might be variation in the strength of an object's color prototypicality (compare, for example, “bone” –‘white’ with “shoe” – ‘black’, ‘red’, etc). We ensured that this variation among objects in our experiment was not correlated with the phrase frequency variable. Specifically, Spanish participants (N = 20) that did not participate in Experiment 1 were asked to provide the first color word that came to mind upon reading the object names used in the Experiment. On the basis of these responses, associated entropy values were computed for each object [33] providing a measure of the degree to which a given object was associated with a particular color. Formula 1 was used to compute the entropy (*H*), where *k* represents the number of different colors given for a particular object, and *p_i_* denotes the proportion of participants that produced a particular color name.
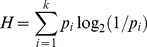
(1)Importantly, these log-transformed entropy values did not correlate with the phrase frequency values (r = −.03), suggesting that the phrase frequency variable was independent from the object's color prototypicality.

Estimates for the frequency of the first and second response, and the frequency of the token phrase were obtained from Google [34]. Token phrase frequencies were obtained using double quotation marks in the query (e.g., “red shoe”).We used the number of hits (“results”) returned by Google as an estimator for an item's lexical frequency. The search algorithm used by Google also takes into account the specific geographic location from which the query is submitted (derived from the computer's IP address). For Experiment 1, the specified location was Santa Cruz de Tenerife, Spain. The advantage of specifying the location while obtaining frequency counts is that the counts were better attuned to the participants in our experiments that live at this location. It should noted these frequency counts are not annotated, and therefore include all homographs. See Table 1 for an overview of the frequency properties of the utterance type variables in Experiment 1 and 2.

**Table 1:** Median (and range between brackets) of the (log) phrase and object frequency of the phrases in Experiment 1 and 2.

	Experiment 1		Experiment 2	
	noun+adjective	noun+noun	noun+adjective	det+noun+adj
Object name freq	16.6 (12.4–19.5)	16.6 (12.4–19.5)	16.2 (12.1–21.4)	16.2 (12.1–21.4)
Phrase freq	8.5 (1.4–13.9)	5.2 (0.00–9.5)	9.5 (4.4–14.1)	9.1 (0.0–14–8)

Note. Det+noun+adj = determiner+noun+adjective; freq = frequency.

The stimuli in noun+adjective condition were created by coloring the outlines of the line drawings using Adobe Photoshop. Stimuli in the noun+noun condition consisted of a black line drawing superimposed on a red line drawing, where the black line drawing corresponded to the first and the red line drawing corresponded to the second response. In total, 100 stimuli (50 noun+adjective, 50 noun+noun) were created. The stimuli in the noun+adjective and noun+noun conditions were presented in a blocked fashion, where the order of the two conditions was counterbalanced across participants. The 50 stimuli for each condition were combined into four pseudo-randomized lists of trials. Responses on consecutive trials were phonologically and semantically unrelated.

**Procedure**: The experimental software was DMDX [35]. Participants wore headphones with attached microphone that provided both voice-key triggering and speech digitization. Prior to the noun+adjective or noun+noun task, the participants were familiarized with the 50 objects corresponding to the first response. In this part all objects were presented in black. On each trial during the familiarization phase, participants saw a fixation cross for 700 ms, followed by the presentation of the picture. After 1000 ms, the object's name appeared beneath the picture for 1000 ms. The presentation of the object's name cued the participant to read the object's name aloud. After a 1000 ms delay the next trial appeared.

In the noun+adjective condition, participants were told they would see the objects from the familiarization part presented in one of ten colors. They were shown the colors and their names, and were told to name each stimulus using a standard Spanish noun phrase (i.e., object followed by color). In the noun+noun condition, participants were told they would see two objects presented on the screen, one superimposed on the other. They were told to first name the object in black followed by the object in red. They were told the names of the ten objects that would appear in red. In both phrase type conditions, the trial structure was identical. On each trial, participants first saw a fixation point for 700 ms, followed by the stimulus for 1500 ms. After a 1000 ms delay the next trial started. Participants were told to respond as fast as possible while producing error-free and fluent speech. The entire experiment lasted about 20 minutes.

### Analysis

Trials on which the participant produced an incorrect first or second response, hesitated or produced any other non-speech sounds were discarded from the analysis (6.3%). Voice-key errors were corrected manually using the CheckVocal software [36]. In addition, outliers were deleted by visual inspection of individual participants' quantile-quantile plots (1.4%). The remaining 2401 trials were analyzed using a mixed effect analysis. For random effects, we considered participants, first response object names, second response object or color names, the phrase, and the interaction of trial by participants as random variables. Model comparisons [37] in which a full model with all variables was iteratively compared to the same model minus one of the random effects variables indicated that only the latter variable (trial by participant) was not justified in the model, and was subsequently removed from the analyses.

The structure of the fixed effect variables in the model included variables that were critical to the question addressed here, as well as a number of control variables that are known to affect latencies in picture naming tasks (e.g., [38]). Critical variables were the frequency of the phrase (phrase frequency), the frequency of the object name corresponding to the first response (first response frequency), the frequency of the color or object name corresponding to the second response (second response frequency), the factor utterance type (noun+adjective versus noun+noun), and the interactions of utterance type and the three frequency variables.

In addition, control variables were the first response's phonological neighborhood size (i.e, [39]), its age of acquisition, familiarity, imageability, and concreteness (all obtained from [40]), as well as the phrase length in phonemes. We also considered the influence of the variable trial (the ordinal position of a stimulus in the course of the experiment). Finally, we considered the influence of three factors related to articulatory processing that have been shown to affect naming latencies [41]: Plosiveness, voicing, and fricativeness. All continuous variables were log-transformed to reduce skewness.

We took into the account the collinearity that existed between the fixed effect predictors by implementing an orthogonalization procedure for those predictors that were strongly correlated (r>.40). Specifically, we regressed phonological neighborhood size of the first response onto phrase length (r = −.52), and regressed both first response and second response frequency onto phrase frequency (r = .41 and r = .64, respectively). We then used the residuals from these three regression analyses as the new values for the phonological neighborhood size, and first and second response frequency, in the regression analyses. The orthogonalized variables correlated highly with the original variables (rs>.77) and therefore the orthogonalization procedure does not compromise their interpretation.

The contribution of the fixed effect factors to the overall explanatory power of the statistical model was evaluated in a backward-stepwise regression analysis. This analysis started with a full model that included all variables, and was trimmed down by excluding variables in a step-by-step fashion. Specifically, a variable was excluded when its regression coefficient did not reach significance (p (MCMC)>.05) in that step of the analysis.

### Results and Discussion

**Figure 1:**
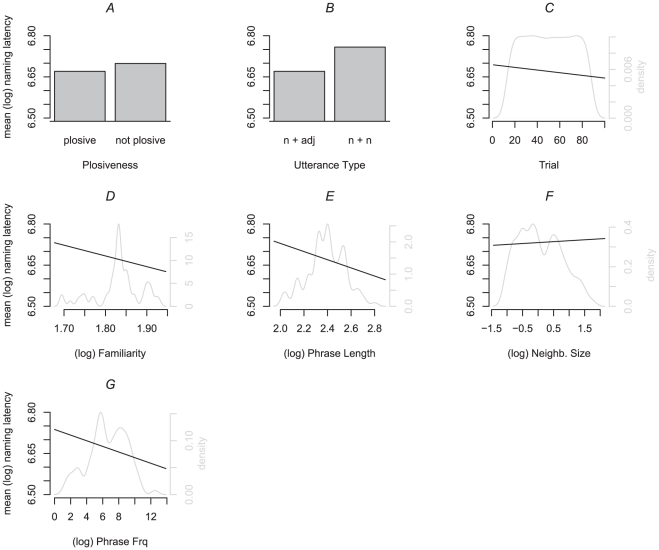
Overview of the partial effects of the fixed effect variables (in black; adjusted for the effects of the other variables), and their density functions (in light gray) in Experiment 1.

Standard deviations for the random intercepts of the by-subject, by-first response, by-second response, by-phrase, and by-observation noise were 0.12, 0.04, 0.02, 0.04, 0.18, respectively. The highest variance inflated factor (VIF) was 1.77, indicating that no problems of collinearity were present in the final model. As can be seen in Table 2 and Figure 1, there was an effect of plosiveness, with faster latencies for items whose onset started with an obstruction versus not (panel A; [41]), and of utterance type, where latencies were faster in the noun+adjective than in the noun+noun condition (panel B). In addition, there were effects of trial, where naming latencies became faster along the course of the experiment (panel C). In line with previous data, there were effects of familiarity, where latencies were faster for object names with high familiarity (panel D; [38]), phrase length, where latencies were faster for longer phrases (panel E), and phonological neighborhood size of the first response, where latencies became slower with increasing neighborhood size (panel F; [39]). Importantly, there was an effect of phrase frequency, where latencies decreased with increasing phrase frequency values (panel G). When object name frequency was removed from the model its statistics were t(2392) = .10, p = .9926.

**Table 2:** Regression coefficients (*β*) with corresponding *t* and *p* values for each of the fixed effect predictors in the regression analyses of Experiment 1.

Predictors	*β (std. error)*	*t* (2393)	*p (MCMC)*
(Intercept)	7.8376 (.2758)	28.41	<.001
Plosiveness	.0288 (.0163)	1.77	<.07
Utterance Type	.0884 (.0186)	4.75	<.001
Trial	−.0005 (.0001)	−3.81	<.001
Familiarity	−.3910 (.1373)	−2.85	<.006
Phrase Length	−.1487 (.0460)	−3.24	<.002
Neighboorhood Size	.0259 (.0094)	2.77	<.008
Phrase Frequency	−.0102 (.0034)	−3.02	<.004

Standard error of the regression coefficient between brackets. Degrees of Freedom associated with the t values between brackets. P values were calculated from Markov chain Monte Carlo confidence intervals using the posterior distribution of the independent variables (Baayen, 2008).

The results of Experiment 1 revealed an effect of phrase frequency in the production of two word phrases. To our knowledge this is the first report of a token phrase frequency effect on naming latencies in language production. The phrase frequency effect did not interact with the factor utterance type, suggesting that phrase frequency affected naming latencies similarly in both noun+adjective and noun+noun phrases. This suggests that the phrase frequency effect is found not only for combinations of words that occur with a high frequency in the language, but also for combinations of words that have a much lower frequency of occurrence in the language. In other words, these findings suggest that the phrase frequency effect is found across the entire frequency continuum [17].

A potential concern is that the phrase frequency effect found here might reflect differences in the recognition of the object in the experimental items. Specifically, it is possible that items corresponding to high frequency phrases contained objects that were easy to recognize, and items corresponding to low frequency phrases had objects that were hard to recognize. However, this interpretation of the frequency effect can be rejected on the basis of the current data, and on the basis of results from previous studies. In the current study we included variables that arguably index object recognition processes such as object familiarity, concreteness, and imageability [38]. However, the correlation between these variables and the variable phrase frequency was small, suggesting that the variable phrase frequency does not index aspects related to object recognition. In addition, many studies show that when frequency effects are found in a picture naming task, they are not found in tasks that assess the recognition of those same pictures [19], [42]–[44]. Finally, a recent study has found that in a delayed, conditional picture naming task, where picture recognition can no longer play a role, latencies are nevertheless sensitive to lexical frequency [45]. This suggests that the phrase frequency effect does not reflect object recognition, and that frequency effects, in general, reflect at least in part the retrieval of linguistic information.

## Experiment 2

In Experiment 2, we attempted to further establish the reliability of the phrase frequency effect in language production by generalizing the effect to a new language: French. In addition, we addressed two additional concerns about the nature of the phrase frequency effect observed in Experiment 1. First, an alternative interpretation of the phrase frequency effect of Experiment 1 is in terms of the semantic integration of the two elements in the stimulus display. Thus, although we were careful in the assignment of the colors to object stimuli in the noun+adjective phrases of Experiment 1 (see Methods), it might be faster to integrate two objects, or a color and an object, when these two elements appear frequently together in the language. In Experiment 2, French participants were asked to produce noun+adjective and determiner+noun+adjective phrases in response to one set of colored objects. If the phrase frequency effect observed in the production of noun+adjective phrases were determined by the ease of semantic integration of the object and the color, one would expect naming latencies in determiner+noun+adjective phrases to be determined by (orthogonalized) noun+adjective phrase frequency, and not by determiner+noun+adjective phrase frequency. This is because the determiner does not impact the ease of integrating an object and a color, and therefore one would expect the frequency of the noun+adjective phrase to determine latencies in both noun+adjective and determiner+noun+adjective phrases. By contrast, if phrase frequency reflects the processing of linguistic information, one would expect naming latencies in noun+adjective and determiner+noun+adjective phrases to be determined by their respective phrase frequencies.

Second, another possible interpretation of the phrase frequency effect observed in Experiment 1 is in terms of the transitional probabilities between individually stored words [46]–[48]. Because transitional probability and phrase frequency are highly correlated in phrases that contain two words (r = .98 in Experiment 1), the three-word determiner+noun+adjective phrases were included to distinguish between phrase frequency and transitional probability.

### Materials and Methods

**Participants**: Forty-four native French speakers, all students at the Université de Provence, participated in the Experiment. Twenty-one participated in the noun+adjective condition, and twenty-three in the determiner+noun+adjective condition.

*Materials*. The materials were taken from Janssen, Alario, and Caramazza ([49], Exp 1). There were fifty-six pictures of objects, selected from the same set as in Experiment 1, and four colors (rouge [red], vert [green], bleu [blue], orange [orange]). Each object was presented twice, each time in a different color, leading to a total of 112 items. For twenty-eight items, the object name and color name had the same phonological onset. For the remaining 84 items the object and color names were phonologically unrelated. As before, we avoided objects with a strong prototypical color, and made sure that assigned colors were non-prototypical. See Table S2 for an overview of the items.

As in Experiment 1, frequency estimates were obtained from Google, this time using Paris, France as the specified location (see Table 1 for a summary). Line-drawings were colored in the same way as in Experiment 1.

Finally, for the determiner+noun+adjective phrases, we considered the transitional probabilities of the individual words in each phrase. Following previous research (e.g., [46]), formula 2 was used to compute the transitional probability (TP) as the bigram frequency between two adjacent words divided by the frequency of the first word.
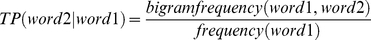
(2)Accordingly, the transitional probability of each token determiner+noun+adjective phrase was computed by the TP of the determiner given the noun multiplied by the TP of the noun given the adjective:
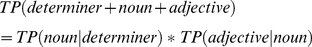
(3)


**Procedure**: The procedure was similar to that used in Experiment 1. Participants took part either in the noun+adjective or in the determiner+adjective+noun condition. Participants in the noun+adjective condition were told to name each colored picture with a standard noun+adjective phrase (e.g., “maison bleue”). Participants in the determiner+noun+adjective condition were instructed to use an indefinite determiner+noun+adjective phrase (e.g., “une maison bleu”). Each naming condition lasted about 20 minutes.

### Analysis

The same exclusion criteria were used as in Experiment 1 (5.2% naming errors, 2.6% outliers). Three items (i.e., “orange” [orange], “jupe” [skirt] and “robe” [dress]) were removed because of high error rates (>20%). 2029 trials were analyzed in the noun+adjective, and 2306 in the determiner+noun+adjective utterance type. Model comparisons led to a random effects structure that included participants and object names as random variables for both utterance types.

The noun+adjective and determiner+noun+adjective conditions were analyzed separately, as different predictors were present in the models for these two utterance types. For the noun+adjective phrases, the critical predictors were the phrase frequency, the frequency of the first response, and the frequency of the second response. For the determiner+noun+adjective phrases, we considered the frequency of the three word phrase, the transitional probability, the frequency of the first response (noun), the frequency of the second response (adjective), the bigram frequency of the determiner+noun phrase, and the bigram frequency of the noun+adjective phrase.

Control variables in both utterance types were the first response's number of phonological neighbors, age of acquisition, familiarity, the picture's image agreement and visual complexity, the phrase length in phonemes, trial, and the three articulatory factors fricativeness, voicing and plosiveness. The articulatory variables were not used in the determiner+noun+adjective utterances, given the lack of onset variation in the determiners. Previous analyses revealed that excluding the phonologically related items did not impact the results (see materials), and were therefore included in the analyses to improve power.

As in Experiment 1, collinearity in the model was reduced by orthogonalization of variables with high correlation (r>.30). For the analyses of both noun+adjective and determiner+noun+adjective utterances, the variable phonological neighborhood size of the first response was decorrelated from the variable phrase length (r = .31), and the variables age of acquisition and first response frequency were decorrelated from the variable phrase frequency (r = −.43, and r = .36, respectively). For the determiner+noun+adjective utterances, the bigram frequencies of the determiner+noun and noun+adjective phrases and the variable transitional probability were decorrelated from the variable phrase frequency (r = .53, r = .70, r = .72, respectively). Other aspects of the analyses were identical to those used in Experiment 1.

### Results and Discussion

**Figure 2:**
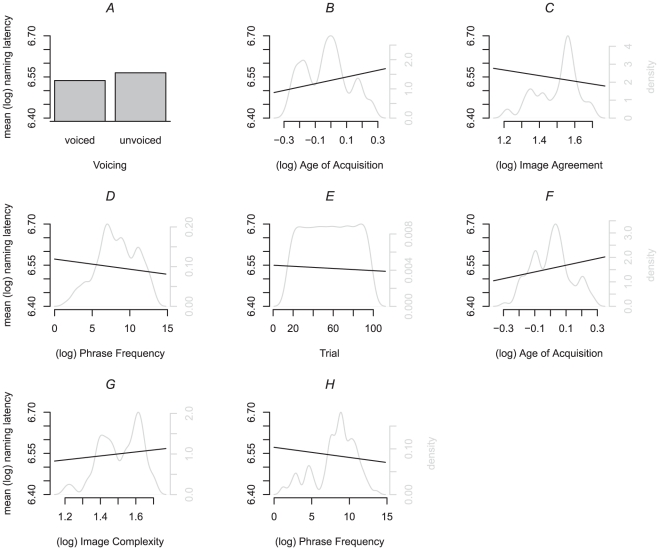
Overview of the partial effects of the fixed effect variables (in black; adjusted for the effects of the other variables), and their density functions (in light gray) for the noun+adjective (panels A–D) and determiner+noun+adjective (panels E–H) in Experiment 2.

**Noun+Adjective utterances**: Standard deviations for the random intercepts of the by-subject, by-picture, and by-observation noise were 0.10, 0.05, and 0.16, respectively. The highest variance inflation factor (VIF) was 1.09, suggesting that no collinearity was present in the model. As can be seen in Table 3 and Figure 2, there was an effect of voicing, where utterances that started with a voiced onset were produced slower than those without a voiced onset (panel A). In addition, in line with other research, there were effects of age of acquisition of the first response, where slower responses were associated with later acquired names (panel B; [50]), and of image agreement, where responses become faster with increasing values of image agreement (panel C; [50]). Importantly, there was an effect of phrase frequency, where latencies decreased with increasing phrase frequency values (panel D). Statistics of the effect of object name frequency when the variable was removed from the model were *t*(2025) = −0.32, *p* = .7314.

**Table 3:** Regression coefficients (*β*) with corresponding *t* and *p* values for each of the fixed effect predictors in the regression analyses of the noun+adjective and determiner+noun+adjective utterances in Experiment 2.

Utterance	Predictors	*β (std. error)*	t*	*p*
Noun+Adjective	(Intercept)	6.7656 (.0889)	76.05	<.001
	Voicing	.0281 (.0153)	1.83	<.06
	AoA	.0822 (.0446)	1.84	<.06
	Image Agreement	−.1025 (.0492)	−2.08	<.04
	Phrase Frequency	−.0075 (.0029)	−2.55	<.009
Determiner+Noun+Adjective	(Intercept)	6.4833 (.0548)	118.60	<.001
	Trial	−.0002 (.0001)	−1.94	<.06
	AoA	.1223 (.0498)	2.45	<.02
	Image Complex	.0724 (.0327)	2.21	<.03
	Phrase Frequency	−.0037 (.0019)	−1.95	<.05

Note. Degrees of freedom for noun+adjective 2024, and for determiner+noun+adjective 2301.

**Determiner+Noun+Adjective utterances**: Standard deviations for the random intercepts of the by-subject, by-picture, and by-observation noise were 0.13, 0.05, and 0.16, respectively. The highest variance inflation factor (VIF) was 1.12, suggesting collinearity was not a problem in the model. As indicated in Table 3 and Figure 2, there was an effect of trial, where responses became faster over the course of the experiment (panel E). In line with other studies, there were effects of age of acquisition, with slower responses for later acquired names (panel F; [50]), and image complexity, which revealed slower responses with more complex images (panel G; [50]). As before, there was an effect of phrase frequency, where latencies became faster with increasing phrase frequency values (panel H). The t- and p-values of the object name frequency effect when it was removed from the model were *t*(2302) = 0.55, *p* = .5580.

These results of Experiment 2 rule out that the phrase frequency effect observed for the noun+adjective phrases reflects the ease of semantic integration of the object and the color. If such were the case, one would have expected noun+adjective phrase frequency to determine naming latencies in both noun+adjective and determiner+noun+adjective phrases, given that the same colored object displays were used for both phrase types, and that the contribution of the determiner is blind to phrase frequency. At odds with this prediction, we observed that naming latencies in the noun+adjective and determiner+noun+adjective phrases were determined by their respective phrase frequencies. It is therefore highly unlikely that the ease of semantic integration played an important role in the results reported here.

**Further Post-hoc tests**: The results of Experiment 1 and 2 revealed that naming pictures with two or three word phrases was sensitive to the frequency of the token phrase, and hence established the robustness of the token phrase frequency effect in language production. To further rule out that the phrase frequency effects were caused by the specific order in which object and color names appear in Romance languages such as Spanish and French, and in order to ensure that the token phrase frequency effects could be found with other stimuli displays, we examined whether the data from two previously published studies on multi-word production revealed effects of phrase frequency. The data from both these studies were analyzed using the methodology of Experiment 1. First, in Janssen and Caramazza ([30], Exp 2A), fifteen native English participants produced adjective+noun phrases in response to depicted colored objects (e.g., ‘red car’). Collapsed across all conditions, naming latencies were predicted by phrase frequency (*β* = −.0135, *t*(2152) = −4.01, *p*<.0003), but not by object name frequency (*β* = .0019, *t*(2152) = 0.36, *p* = .7358). Likewise, in Janssen, Schiller, and Alario [51], thirty-two native Dutch participants produced adjective+noun or determiner+adjective+noun phrases in response to big or small depicted objects (e.g., ‘kleine auto’ [small car], ‘groot paard’ [big horse]). The size of the presented object denoted the adjective. Objects could be named in singular or plural. Collapsed across all conditions, naming latencies in adjective+noun phrases were predicted by phrase frequency (*β* = −.0071, *t*(1207) = −1.97, *p*<.05), but not by object name frequency (*β* = −.0027, *t*(1207) = −0.66, *p* = .4782). Likewise, naming latencies in determiner+noun+adjective phrases (taking into account orthogonalized bigram frequencies) were predicted by phrase frequency (*β* = −.0076, *t*(1492) = −3.85, *p*<.0003), but not by object name frequency (*β* = −.0007, *t*(1492) = −0.18, *p* = .8556).

The effect of token phrase frequency is thus found in various languages (Spanish, French, English, Dutch), with various types of stimulus displays (colored objects, two superimposed objects, big and small objects), and with phrases consisting of two or three words (noun+adjective, noun+noun, adjective+noun, determiner+noun+adjective, determiner+adjective+noun). These data firmly establish the robustness of the effect of token phrase frequency in the production of multi-word utterances.

## Discussion

We examined whether the frequency of a multi-word phrase determined naming latencies in a language production context. In Experiment 1 and 2, participants produced two- or three-word phrases in response to stimulus displays depicting two superimposed objects or colored objects. The token phrase frequency and the frequency of the object noun in the multi-word phrases were independently manipulated. The results from both experiments, and from post-hoc analyses of two published data sets revealed that naming latencies in the multi-word phrases were affected by the token phrase frequency. No effect of object name frequency was observed.

These results are the first observation that phrase frequency affects naming latencies in a language production context. This finding is in line with previous studies in the field of language production that have examined the impact of phrase frequency on measures of phonological reduction [26], and agrees with findings from the field of language comprehension [17], [20]–[25]. For example, as discussed in the Introduction, Arnon and Snider [17] found that recognition times of multi-word phrases were sensitive to the frequency with which the phrases appeared in the language. These authors also found that the phrase frequency effects were observed at low, middle, and high frequency points along the frequency continuum. The current results are consistent with this observation. They revealed phrase effects for utterance types that are high frequent (noun+adjective) and low frequent (noun+noun) in the language, suggesting that phrase frequency effect in language production are also found across the entire frequency spectrum. The overall conclusion that follows from these data is that the observation of a phrase frequency effect is a general phenomenon that is not tied to a specific modality of language use.

The results reported here are problematic for the words and rules view that assumes that the language system is sensitive to the distribution of linguistic information at rather restricted grain-sizes [3], [4]. This model assumes that there are representations for morphologically simple forms that are stored in the lexicon, and that there are rules that combine these simple forms into complex words and multi-word phrases. Accordingly, the model assumes that language users should be sensitive only to the distribution of morphologically simple forms in the language environment, and therefore predicts a frequency effect for morphologically simple forms, but not for multi-word phrases. At odds with these predictions, naming latencies in our experiments were sensitive to multi-word phrase frequency. As discussed in the Introduction, traditional models of language production [27], [28] that adhere to the words and rules view are also challenged by the current results.

These results are consistent with a class of models that we have labeled emergentist in the Introduction. These models include connectionist [5]–[7], usage-based [8]–[11], and exemplar-based [12]–[15] models of language processing. A common aspect of these models is their emphasis on the role of experience in the language system, where a language's grammar emerges from the experience a user has with language. Emergentist models therefore provide direct motivation for the assumption that linguistic forms of grain-sizes larger than morphologically simple forms will be entrenched in memory. As such models of this type provide a natural explanation of the results observed here. Specifically, within the context of these models, the phrase frequency effects observed here reflect the sensitivity of the language system to the distribution of linguistic information at grain-sizes beyond morphologically simple forms. In addition, the finding in Experiment 1 that the phrase frequency effect was found for both high frequent and low frequent phrase types suggests that the entrenchment of complex linguistic forms in memory does not need to pass a certain frequency threshold. Even with very little exposure, as was the case in the Spanish noun+noun phrases, token linguistic forms become entrenched in memory.

Although the results indeed suggest a language system that is sensitive to the distribution of information at grain-sizes that include multiple words, it is unclear by which specific mechanism the observed multi-word phrase frequency effects come about. We discuss three possible alternatives. First, it is possible that the phrase frequency effect reflects the transitional probability between individually stored words [46]–[48], where words that appear in high frequency phrases have higher transitional probabilities than words in low frequency phrases. However, there are both empirical and theoretical reasons why this hypothesis is unlikely. First, our results did not show a role for transitional probability that is independent from phrase frequency. Specifically, analyses of the determiner+noun+adjective phrases of Experiment 2 included orthogonalized variables of phrase frequency and transitional probability, and revealed that naming latencies were affected by the phrase frequency and not transitional probability. This suggests that transitional probabilities do not play a role in the production of multi-word phrases beyond that played by phrase frequency. More generally, the explanation of a phrase frequency effect in terms of transitional probabilities rests on the assumption that transitional probability between individual words correlates with the frequency of a phrase as a whole. This seems a plausible assumption for two-word phrases, but this assumption becomes increasingly implausible when considering longer phrases that contain three or four words. In short, it seems unlikely that the phrase frequency effect reflects the transitional probabilities between individually stored words.

A second possibility is that the phrase frequency effect reflects the connection weights between input and output units in a connectionist architecture. It is well known that basic connectionist models are able to generate both part- and surface-frequency effects in the comprehension of morphologically complex words [52]. More recently, Baayen et al. [5] have shown that a two-layer connectionist model based on discriminative learning can simulate empirically observed multi-word phrase frequency effects. Thus, a second possibility is that the phrase frequency effect observed here reflects the connection weights between input and output representations. One way in which this proposal may be further evaluated is by considering its predictions regarding the presence of part- and surface-frequency effects in the production of multi-word phrases. Specifically, models of this kind assume that multi-word phrases are stored fully decomposed, and therefore predict that naming latencies to multi-word phrases should be sensitive to both part- and surface-frequency of the multi-word phrase. However, this prediction is not borne out by the results presented here. Specifically, our results revealed that production latencies were sensitive to the frequency of the multi-word phrase, but not to the frequency of the object name in the phrase. Thus, it is unclear whether basic connectionist architectures such as the ones described here provide a coherent account of our results.

A final possibility is that the phrase frequency effects reflect the retrieval of a multi-word phrase represented as a single holistic chunk. In this proposal, the observed phrase frequency effect may be explained by assuming that the frequency of a phrase is reflected in a representation's activation level, and that retrieval times of representations are determined by their activation levels. Under such circumstances, one would expect retrieval times to be determined by the frequency of the multi-word phrase, but not by the frequency of its component parts. Note that within the context of this proposal, the effects of object age of acquisition and familiarity observed in the experiments above could reflect semantic level processes [38], [53]. The view that multi-word phrases are stored holistically resonates well with results from other studies on multi-word production. Specifically, researchers that have examined the impact of phrase frequency on phonological reductions have argued that repeated exposure to multi-word phrases leads to their holistic storage in memory [26].

In addition, the assumption that multi-word phrases are represented by a single holistic chunk is supported by two independent lines of research. First, studies examining the production of morphologically complex compound words have concluded that words of this type are stored holistically [19]. This conclusion was based on the observation that naming latencies to pictures with compound names (e.g., ‘doghouse’) were sensitive to the surface frequency of the compound word, but not to the frequency of its component parts, parallel to the results observed here. Second, the assumption of holistic representations of complex words and multi-word phrases fits with insights from the field of language acquisition. Research in this field has shown that early grammar primarily relies on the use of unparsed holistic phrases (i.e., holophrases such as ‘lemme see’; [11]). One possibility is that the use of such holistic representations does not end in early childhood, but that its use continues throughout adulthood. The assumption that complex words and multi-word phrases are stored holistically does not mean that individual words are not also stored, nor that effects of its component parts cannot be detected experimentally. Within the emergentist literature it is assumed that holistic phrases form the basis for the development of abstract grammatical constructions through the application of general cognitive mechanisms such as categorization and analogy [8]–[11]. In addition, a large literature has demonstrated that the component parts of morphologically complex words affect word recognition times [18], suggesting that the detection of the effects for component parts of complex words is context dependent. To summarize, the current results fit within the general assumptions of the models proposed in the emergentist framework. Within this context we have discussed three alternative accounts of our data. Although we cannot rule out explanations in terms of transitional probabilities or basic connectionist architectures, our preferred explanation is that multi-word phrases are represented by a single holistic chunk.

A surprising aspect of our data is that the effect of phrase frequency was found in the absence of any effects of the frequency of the component parts. Thus, in both Experiment 1 and 2, naming latencies were determined by the frequency of the phrase, but not by the frequency of the first or second response, or by any of the bigram frequencies. This is surprising in light of previous studies where an effect of object name frequency was found in the production of multi-word phrases. For example, in Alario et al. [29], naming latencies were faster for multi-word utterances that contained a high frequency object name compared to utterances that contained a low frequency object name. There are at least three possible explanations for this state of affairs. First, the frequency counts we obtained from Google might be less accurate than those from more traditional databases, and hence, our manipulation of frequency might have been noisy. We addressed this issue by re-analyzing the data from Experiment 1 and 2, but now using frequency counts obtained from SubtLex [54] for Experiment 1, and Lexique [55] for Experiment 2. However, this did not change the results – no effect of object name frequency emerged. Second, it is possible that the range of frequencies tested in our experiments was too narrow to reveal effects. However, this would mean that the frequency manipulation was too narrow to be detected in Experiment 1, 2 and in the post-hoc analyses – this seems unlikely. In addition, the ranges in our experiments were 1–1381 (SubtLex, Experiment 1), and 1–491 (Lexique, Experiment 2), which do not seem to differ substantially from the 1–662 range reported by Alario et al. A final possible explanation is that in our experiments object name and phrase frequency were independently manipulated, while in Alario et al., object name, but not phrase frequency was manipulated. Given the natural correlation between object name frequency and phrase frequency, it is possible that the object name frequency effect observed by Alario et al. is in fact a phrase frequency effect in disguise. If such were the case, one could conclude that naming latencies of multi-word phrases are determined by the frequency of the phrase that is being produced, and not by the frequency of the component parts of the phrase. Such a conclusion would resonate well with conclusions reached on the basis of the comprehension and production of morphologically complex words [18], [19].

To conclude, the current study presents the first observation that production latencies are sensitive to the frequency of multi-word phrases. These data are in line with those observed in visual word comprehension and thereby generalize this effect across different modalities of language use. The phrase frequency effect is at odds with the words and rules view, and with most current models of language production that endorse this view of the organization of the mental lexicon [3], [4], [27], [28]. Instead, the finding that speakers are sensitive to the frequency with which a multi-word sequence occurs in the language is in line with the emergentist view of the language system [5]–[15]. This view assumes that the language system is sensitive to the distribution of linguistic information at grain-sizes beyond individual words. Within such a system, the observed phrase frequency effect might reflect transitional probabilities between individually stored words [46]–[48], the connection weights between low-level input and higher level output representations [5]–[7], or the retrieval of multi-word chunks from memory [20], [26]. A pertinent question for future research would be to distinguish between these three possible explanations of the phrase frequency effects observed here.

## Supporting Information

Table S1 **Overview of the objects and colors used in the noun+adjective and noun+noun utterances of** **Experiment 1** **.**
(DOC)

Table S2 **Overview of the objects and colors used in the noun+adjective and determiner+noun+adjective utterances of** **Experiment 2** **.**
(DOC)
