# Supplementary material for: Phrase Frequency Effects in Language Production
Source: PLoS One. 2012 Mar 27;7(3):e33202. doi: 10.1371/journal.pone.0033202 (PMC3314013; doi:10.1371/journal.pone.0033202)
Supplement: Table S1 — Overview of the objects and colors used in the noun+adjective and noun+noun utterances of Experiment 1 . (DOC) [file pone.0033202.s001.doc]

Table S1. Items Experiment 1.

| Noun + adjective | Noun + noun |
| --- | --- |
| ancla gris (gray anchor) | ancla güante (anchor mitten) |
| arpa gris (gray harp) | arpa güante (harp mitten) |
| bombero rosa (pink fireman) | bombero rana (fireman frog) |
| botella marron (brown bottle) | botella mujer (bottle woman) |
| bufanda rosa (pink scarf) | bufanda rana (scarf frog) |
| caja verde (green box) | caja volcán (box volcano) |
| calcetín amarillo (yellow sock) | calcetín anillo (sock ring) |
| cama azul (blue bed) | cama avión (bed airplane) |
| campana gris (gray bell) | campana güante (bell mitten) |
| candado rojo (red lock) | candado rodillo (lock rolling pin) |
| casa verde (green house) | casa volcán (house volcano) |
| cepillo negro (black brush) | cepillo niño (brush boy) |
| clavo naranja (orange nail) | clavo niña (nail girl) |
| clip negro (black paperclip) | clip niño (paperclip boy) |
| collar azul (blue necklace) | collar avión (necklace airplane) |
| corazón morado (purple heart) | corazón martillo (heart hammer) |
| cuchara marron (brown spoon) | cuchara mujer (spoon woman) |
| escoba naranja (orange broom) | escoba niña (broom girl) |
| espejo rojo (red mirror) | espejo rodillo (mirror rolling pin) |
| flecha marron (brown arrow) | flecha mujer (arrow woman) |
| fuente azul (blue fountain) | fuente avión (fountain airplane) |
| girafa naranja (orange giraffe) | girafa niña (giraffe girl) |
| globo verde (green globe) | globo volcán (globe volcano) |
| helicóptero morado (purple helicopter) | helicóptero martillo (helicopter hammer) |
| hoja rosa (pink leaf) | hoja rana (leaf frog) |
| hueso naranja (orange bone) | hueso niña (bone girl) |
| kanguro morado (purple kangaroo) | kanguro martillo (kangaroo hammer) |
| lápiz verde (green pencil) | lápiz volcán (pencil volcano) |
| libro amarillo (yellow book) | libro anillo (book ring) |
| mochila azul (blue bag) | mochila avión (bag airplane) |
| moneda rosa (pink coin) | moneda rana (coin frog) |
| ojo verde (green eye) | ojo volcán (eye volcano) |
| pastel marron (brown cake) | pastel mujer (cake woman) |
| perro gris (gray dog) | perro güante (dog mitten) |
| piano rojo (red piano) | piano rodillo (piano rolling pin) |
| pie rosa (pink foot) | pie rana (foot frog) |
| plato amarillo (yellow plate) | plato anillo (plate ring) |
| reloj morado (purple watch) | reloj martillo (watch hammer) |
| sandwích amarillo (yellow sandwich) | sandwích anillo (sandwich ring) |
| semáforo negro (black stoplight) | semáforo niño (stoplight boy) |
| serrucho azul (blue saw) | serrucho avión (saw airplane) |
| silla gris (gray chair) | silla güante (chair mitten) |
| tambor rojo (red drum) | tambor rodillo (drum rolling pin) |
| taza naranja (orange cup) | taza niña (cup girl) |
| teléfono negro (black telephone) | teléfono niño (telephone boy) |
| tenedor amarillo (yellow fork) | tenedor anillo (fork ring) |
| trompo morado (purple top) | trompo martillo (top hammer) |
| ventana marron (brown window) | ventana mujer (window woman) |
| volante negro (black steering wheel) | volante niño (steering wheel boy) |
| zapato rojo (red shoe) | zapato rodillo (shoe rolling pin) |
