# Supplementary material for: Phrase Frequency Effects in Language Production
Source: PLoS One. 2012 Mar 27;7(3):e33202. doi: 10.1371/journal.pone.0033202 (PMC3314013; doi:10.1371/journal.pone.0033202)
Supplement: Table S2 — Overview of the objects and colors used in the noun+adjective and determiner+noun+adjective utterances of Experiment 2 . (DOC) [file pone.0033202.s002.doc]

Table S2. Items Experiment 2.

| Noun + adjective | Determiner + noun + adjective |
| --- | --- |
| pomme bleu (blue apple) | une pomme bleu |
| pomme rouge (red apple) | une pomme rouge |
| flèche bleu (blue arrow) | une flèche bleu |
| flèche rouge (red arrow) | une flèche rouge |
| ballon bleu (blue ball) | un ballon bleu |
| ballon vert (green ball) | un ballon vert |
| barbecue bleu (blue barbecue) | un barbecue bleu |
| barbecue vert (green barbecue) | un barbecue vert |
| panier bleu (blue basket) | un panier bleu |
| panier rouge (red basket) | un panier rouge |
| ours orange (orange bear) | un ours orange |
| ours rouge (red bear) | un ours rouge |
| banc bleu (blue bench) | un banc bleu |
| banc vert (green bench) | un banc vert |
| oiseau orange (orange bird) | un oiseau orange |
| oiseau rouge (red bird) | un oiseau rouge |
| bouteille bleu (blue bottle) | une bouteille bleu |
| bouteille vert (green bottle) | une bouteille vert |
| bouton bleu (blue button) | un bouton bleu |
| bouton vert (green button) | un bouton vert |
| cactus orange (orange cactus) | un cactus orange |
| cactus rouge (red cactus) | un cactus rouge |
| bougie bleu (blue candle) | une bougie bleu |
| bougie vert (green candle) | une bougie vert |
| voiture orange (orange car) | une voiture orange |
| carotte bleu (blue carrot) | une carotte bleu |
| carotte rouge (red carrot) | une carotte rouge |
| voiture vert (green car) | une voiture vert |
| chat bleu (blue cat) | un chat bleu |
| chat rouge (red cat) | un chat rouge |
| chaise bleu (blue chair) | une chaise bleu |
| chaise rouge (red chair) | une chaise rouge |
| vache orange (orange cow) | une vache orange |
| vache vert (green cow) | une vache vert |
| bureau bleu (blue desk) | un bureau bleu |
| bureau vert (green desk) | un bureau vert |
| robe bleu (blue dress) | une robe bleu |
| robe rouge (red dress) | une robe rouge |
| oreille orange (orange ear) | une oreille orange |
| oreille rouge (red ear) | une oreille rouge |
| éléphant bleu (blue elephant) | un éléphant bleu |
| éléphant rouge (red elephant) | un éléphant rouge |
| oeil orange (orange eye) | un oeil orange |
| oeil rouge (red eye) | un oeil rouge |
| robinet bleu (blue dresser) | un robinet bleu |
| robinet rouge (red dresser) | un robinet rouge |
| plume orange (orange feather) | une plume orange |
| plume rouge (red feather) | une plume rouge |
| drapeau bleu (blue flag) | un drapeau bleu |
| drapeau vert (green flag) | un drapeau vert |
| fourchette bleu (blue fork) | une fourchette bleu |
| fourchette vert (green fork) | une fourchette vert |
| renard bleu (blue fox) | un renard bleu |
| renard rouge (red fox) | un renard rouge |
| verre orange (green glass) | un verre orange |
| verre vert (green glass) | un verre vert |
| coeur bleu (blue heart) | un coeur bleu |
| coeur vert (green heart) | un coeur vert |
| cheval bleu (blue horse) | un cheval bleu |
| cheval vert (green horse) | un cheval vert |
| maison bleu (blue house) | une maison bleu |
| maison vert (green house) | une maison vert |
| igloo orange (orange igloo) | un igloo orange |
| igloo rouge (red igloo) | un igloo rouge |
| clef bleu (blue key) | une clef bleu |
| clef vert (green key) | une clef vert |
| couteau bleu (blue knife) | un couteau bleu |
| couteau vert (green knife) | un couteau vert |
| échelle orange (orange ladder) | une échelle orange |
| échelle vert (green ladder) | une échelle vert |
| lampe orange (orange lamp) | une lampe orange |
| lampe vert (green lamp) | une lampe vert |
| ampoule orange (orange lightbulb) | une ampoule orange |
| ampoule vert (green lightbulb) | une ampoule vert |
| oignon orange (orange onion) | un oignon orange |
| oignon rouge (red onion) | un oignon rouge |
| orange orange (orange orange) | une orange orange |
| orange rouge (red orange) | une orange rouge |
| palmier orange (orange plam tree) | un palmier orange |
| palmier rouge (red palm tree) | un palmier rouge |
| crayon orange (orange pencil) | un crayon orange |
| crayon vert (green pencil) | un crayon vert |
| pipe orange (orange pipe) | une pipe orange |
| pipe vert (green pipe) | une pipe vert |
| lapin orange (orange rabbit) | un lapin orange |
| lapin vert (green rabbit) | un lapin vert |
| râteau bleu (blue rake) | un râteau bleu |
| râteau rouge (red rake) | un râteau rouge |
| règle bleu (blue ruler) | une règle bleu |
| règle rouge (red ruler) | une règle rouge |
| vis orange (orange screw) | une vis orange |
| vis vert (green screw) | une vis vert |
| requin bleu (blue shark) | un requin bleu |
| requin rouge (red shark) | un requin rouge |
| jupe orange (orange skirt) | une jupe orange |
| jupe vert (green skirt) | une jupe vert |
| valise orange (orange luggage) | une valise orange |
| valise vert (green luggage) | une valise vert |
| soleil orange (orange sun) | un soleil orange |
| soleil rouge (red sun) | un soleil rouge |
| orteil orange (orange toe) | un orteil orange |
| orteil rouge (red toe) | un orteil rouge |
| feu orange (orange stoplight) | un feu orange |
| feu rouge (red stoplight) | un feu rouge |
| vase orange (orange vase) | un vase orange |
| vase vert (green vase) | un vase vert |
| violon orange (orange violin) | un violon orange |
| violon vert (green violin) | un violon vert |
| roue bleu (blue wheel) | une roue bleu |
| roue rouge (red wheel) | une roue rouge |
| sifflet orange (orange whistle) | un sifflet orange |
| sifflet rouge (red whistle) | un sifflet rouge |
